# Factors associated with multiple births in the United Kingdom during COVID-19: A cross-sectional survey

**DOI:** 10.1371/journal.pone.0352340

**Published:** 2026-06-26

**Authors:** Yan-Shing Chang, Jessica Jones, Seo Ah Hong, Li-Yin Chien

**Affiliations:** 1 Florence Nightingale Faculty of Nursing, Midwifery & Palliative Care, King’s College London, London, United Kingdom; 2 Department of Public Health Administration, Faculty of Public Health, Mahidol University, Bangkok, Thailand; 3 Institute for Health and Society, Hanyang University, Seoul, Republic of Korea; 4 Institute of Community Health Care, College of Nursing, National Yang Ming Chiao Tung University, Taipei, Taiwan; National Research Centre, EGYPT

## Abstract

**Background:**

Women with multiple births may encounter many challenges compared to those with singleton births, especially during the COVID-19 pandemic. This is the first study aiming to investigate the factors associated with multiple births during COVID-19 in the United Kingdom.

**Methods:**

A cross-sectional online survey was carried out between July and November 2021 with women who were up to 6 months postpartum. Sociodemographic, obstetric, and COVID-19-related factors, and infant feeding and support factors, were employed to assess the association with multiple births. Descriptive statistics and bivariate and multivariable analyses were performed.

**Results:**

Of 858 eligible participants, 53 had multiple births. Bivariate analysis showed that although socioeconomic and COVID-19-related factors were not associated, preterm birth, Neonatal Intensive Care Unit admission, birthweight, mode of birth, postnatal depression, infant feeding practices, impact of COVID-19 on baby fed with expressed breast milk, no support for infant feeding, infant feeding support from online group were associated with multiple births. In the multiple logistic regression, multiple births were significantly associated with infant formula use (AOR 6.17, 95% CI 2.29–16.57), preterm birth (AOR 5.83, 95% CI 1.99–17.11), caesarean birth (AOR 2.97, 95% CI 1.27–6.97), and birthweight (<2.5 kg: AOR 13.16, 95% CI 4.31–40.20;  > 3.5 kg: AOR 0.11, 95% CI 0.02–0.51).

**Conclusions:**

Participants with multiple births experienced higher obstetric risks and were more likely to feed with infant formula. These findings underscore the need for routine integration of lactation specialists and the expansion of remote support strategies.

## Introduction

A multiple pregnancy is when a gestation results in multiple foetuses [1]. Multiple births have been increasing since the 1980s, with the rate in England and Wales having increased from 9.8 per 1000 of all maternities in 1980 to 14.4 per 1000 in 2020 [2]. Factors including an increased use of assisted conception, a trend to having children later in life and a higher survival rate of premature babies may contribute to this increase [3].

Multiple births are regarded as high risk with a higher likelihood of obstetric complications and poorer mental health compared to singleton births [1]. For example, women with multiple pregnancies were more likely to have caesarean birth even though vaginal birth was planned [4,5], preterm birth [6], and babies with low birthweight [7,8]. Additionally, they had worse postnatal mental health outcomes compared to those with singleton births [9]. They were less likely to breastfeed [10] which may be due to the physical challenges of caring for and feeding more than one baby [11], or also be a result of a perception that breastmilk alone was not enough to sustain the nutritional requirements of twins [12]. Despite the additional support needed for breastfeeding in multiple births, very few studies on breastfeeding interventions included women with multiple births [13].

The high-risk nature of multiple pregnancies was compounded by COVID-19, creating additional challenges. For example, a study in India found that COVID- 19 infection and multiple pregnancies increased women’s risk of preterm birth compared to singleton pregnancies [14]. Women who tested COVID-19 positive or were symptomatic were recommended to breastfeed by the World Health Organisation (WHO) while taking standard COVID precautions such as wearing a mask and hand washing or, in the case of severe illness, expressing milk for feeding [15]. However, Jonsdottir et al’s [16] research conducted in 2020−21 found that compared to twins born at term, parents of late preterm twins faced distinctive challenges which increased the likelihood of breastfeeding cessation after one month postpartum. Lack of access to support via social networks and health services had negative impact on both breastfeeding practices and maternal mental health during COVID-19 [17]. With more than one baby to feed and look after simultaneously, lack of access to social and health provider support due to COVID-19 could negatively affect those with multiple births more than singleton births.

Considering the vulnerability of families with multiple births and the adverse impact COVID-19 has on the perinatal outcomes, research during COVID-19 is needed to inform practices to support this group and prepare for future pandemics. Our study aimed to establish the factors, including infant feeding practices, support, and obstetric factors, associated with multiple births and to address the gap that no such research had been conducted during the pandemic in the United Kingdom (UK).

## Methods

### Study design and participants

The cross-sectional online survey was carried out in the UK between July 2021 and November 2021. A convenience sampling strategy was used to recruit women who were up to 6 months postpartum living in the UK, aged 18–49 years old, and able to fill in the online survey in English. Convenience sampling was suitable to address the aim of the study due to its ease of access to potential participants at a low cost, and less time consuming. However, its self-selected nature means that the sample may not represent the intended population, may present selection bias and limit generalizability [18].

A message about the study with the survey weblink was posted on social media (e.g., Twitter, Facebook) and online mothers and babies groups to recruit participants. A similar message about the study with the survey weblink was sent to contacts at children centres, not-for-profit organisations, and personal and professional networks for them to pass on to potential participants, and/or advertise the study on their newsletters, social media, and webpages. As online convenience sampling was used, sample size was not calculated.

### Data collection

Anonymous data were collected using an online Google Form. Two midwifery and nursing professionals, and five postnatal women, including those with multiple births pretested the questionnaire which was then revised to increase clarity. Online informed consent was completed by all participants before proceeding to the survey questions. The Psychiatry Nursing and Midwifery Research Ethics Subcommittee at King’s College London (HR/DP-20/21–22651, RESCM-20/21–22651) granted ethical approval of the study.

### Measures of variables

‘Multiple births’ was the dependent variable and was obtained by the question, ‘Is your [youngest] baby one of twins, triplets or other multiple birth? (‘No, singleton’, ‘Yes, twin, triplets or other multiple birth’). The independent variables were employed based on literature review; (a) socio-demographic factors (child age (1-2, 3-4, 5-6 months of age), maternal age (18-29, 30-39, 41-49 years), maternal education, marital status. working status, residence (urban or rural), ethnicity, and COVID-19-related factors), (b) obstetric factors (preterm/term birth, admission to Neonatal Intensive Care Unit (NICU), birthweight (<2.5kg, 2.5-3.5kg, >3.5kg), mode of birth), and (c) Infant feeding and support factors, (d) mental health factor which was measured by Edinburgh Postnatal Depression Scale (EPDS) to assess participants’ self-reported mental health in the last seven days. EPDS consists of 10 items with Likert scales (scoring 0–3). A total score ranges from 0 to 30 [19,20]. A cut-off point of 13 or more EPDS score was classified as having postnatal depression.

Self-reported and not verified COVID-19 related factors consisted of (a) COVID-19 infection (yes or no), (b) COVID-19 vaccination uptake (yes or no), (c) worry about being (re)infected by coronavirus (‘not at all worried’, ‘a little worried’, ‘moderately worried’, ‘very worried’, ‘extremely worried’), and (d) impact of COVID-19 on food insecurity before and during COVID-19, with two questions: (1) ‘Did you ever run out of food before the end of the month or cut down on the amount you ate to feed others in 2019 BEFORE COVID-19?’ and (2) ‘Did you ever run out of food before the end of the month or cut down on the amount you ate to feed others DURING COVID-19 in 2020-2021?’. The answers were coded into four categorized: ‘no change: insecure’, ‘worse (secure to insecure)’, ‘better (insecure to secure)’ and ‘no change: secure’.

Infant feeding factors included (a) breastfeeding intention during pregnancy (b) baby was fed directly from breast in the last 24 hours, (c) baby was fed expressed breastmilk in the last 24 hours, (d) baby was fed infant formula in the last 24 hours, (e) baby was fed complementary foods in the last 24 hours, (f) completed stopped breastfeeding and giving expressed breastmilk. These measures of various infant feeding practices would help understand if there were any differences in feeding practices between those with multiple and singleton births. Impact of COVID-19 on infant feeding included (a) impact of COVID-19 on baby fed directly on breast which was asked with options of response (1) not intend to feed, (2) shorter than I intended, (3) the same duration as I intended, and (4) longer than I intended, (b) impact of COVID-19 on baby fed expressed breastmilk which was asked with options of response (1) not intend to feed, (2) shorter than I intended, (3) the same duration as I intended, and (4) longer than I intended. Participants’ belief of breastfeeding relating COVID-19 was also asked. A total of six questions (e.g., A breastfeeding mother who is confirmed or suspected to have COVID-19 should always wear a face mask when breastfeeding) were developed for beliefs of breastfeeding in relation to COVID-19 infection prevention and control measures based on WHO’s [15] breastfeeding Questions & Answers. ‘Disagree’, ‘uncertain’ and ‘agree’ were the answer options. A maximum total is 12 and minimum is 0. A higher score shows a more positive belief of breastfeeding.

The variables of support, which included support methods used during COVID-19 such as remote contacts, consisted of (a) from whom support was received for postnatal infant feeding (mark all that apply) (‘I receive no support for infant feeding’, ‘from healthcare professionals’, ‘from spouse/partner, friend or relative’, ‘from online support group (e.g., Facebook)’, and ‘other’ (b) methods of contacts with healthcare professionals for postnatal breastfeeding support (mark all that apply) (‘never’, ‘in person’, ‘by phone’, ‘video’, and ‘other’; (c) experience of any difficulties when receiving breastfeeding support via video contact among those who made a video contact (Mark all that apply) (‘no difficulties’, ‘supporter unable to clearly see the baby’s latch on my breast’, ‘supporter was unable to hear me well’, ‘could not hear the supporter well’, ‘could not see the supporter well’, ‘could not operate the device and breastfeed at the same time’, and ‘other’), and (d) social support assessed using ‘Maternity Social Support Scale (MSSS), a 6-item self-report scale [21], and coded into two categories: low/medium social support (<25 scores), and high social support (25–30 scores).

### Data analysis

Data analyses were carried out using SAS statistical software package version 9.3 (SAS Institute Inc., Cary, NC, USA). For descriptive statistics, numbers and percentages were presented for the categorical variables, and mean and standard deviation (SD) were for the continuous variables. Chi-square test or fisher’s exact test was performed to test the association between ‘multiple births (yes or no)’ (dependent variable) and the independent variables in the bivariate analyses. The independent variables with p-value <0.05 in the bivariate analysis were used in the multiple logistic regression to determine associated factors with multiple births. The strength of the associations between the independent variables and multiple births was examined by the use of adjusted odds ratio (AOR) with 95% confidence interval (CI).

## Results

Of 877 postnatal women who filled in the survey, 858 were eligible, with 805 (93.8%) having singleton births and 53 (6.2%) having multiple births. More than 50% of participants with singleton or multiple births were between 30−39 years old, had an undergraduate or higher degree, were married, lived in urban areas, were white ethnicity, and were multiparous. In addition, the majority reported that they had not tested COVID-19 positive, and had at least one doze of COVID-19 vaccine, and were somewhat or very worried about getting infected with the coronavirus, but did not experience any impact of COVID-19 on food insecurity (Table 1). Bivariate analysis revealed that socio-demographic factors and COVID-19 related factors were not associated, while obstetric factors such as preterm birth, NICU admission, birthweight, mode of birth, and postnatal depression were statistically significantly associated with multiple births (Table 1).

**Table 1:** Association between singleton/multiple births and socio-demographic, obstetric and COVID-19 related factors.

	Total		Singleton Births		Multiple Births		p-value
	n	%	n	%	n	%	
	858	100	805	93.8	53	6.2	
**Sociodemographic factors**							
Age of child							
1-2 months	300	35.0	280	34.8	20	37.7	0.5462
3-4 months	302	35.2	287	35.7	15	28.3	
5-6 months	256	29.8	238	29.6	18	34.0	
Maternal age							
18-29 yrs	186	21.7	180	22.4	6	11.3	0.1249
30-39 yrs	619	72.1	577	71.7	42	79.3	
41-49 yrs	53	6.2	48	6.0	5	9.4	
Education of mother (undergraduate degree or higher)^†^	672	78.4	628	78.1	44	83.0	0.4001
Marital status (married)	840	97.9	788	97.9	52	98.1	0.9118
Working status (yes)	31	3.6	30	3.7	1	1.9	0.4869
Residence (Urban)	541	63.1	506	62.9	35	66.0	0.6422
Ethnicity (white)	826	96.3	775	96.3	51	96.2	0.9861
**Obstetric factors**							
Parity (First baby)	393	45.8	370	46.0	23	43.4	0.7164
Preterm birth(yes)	66	7.7	32	4.0	34	64.2	**<.0001**
NICU admission (yes)	80	9.3	58	7.2	22	41.5	**<.0001**
Birthweight							
Less than 2.5 kg	51	5.9	18	2.2	33	62.3	**<.0001**
2.5-3.5 Kg	350	40.8	332	41.2	18	34.0	
More than 3.5 Kg	457	53.3	455	56.5	2	3.8	
Mode of birth (caesarean birth)	302	35.2	266	33.0	36	67.9	**<.0001**
Postnatal depression (13 or higher)	228	26.6	221	27.5	7	13.2	**0.0230**
**COVID-19-related factors**							
Ever tested as COVID-19 positive (yes)	127	14.8	117	14.5	10	18.9	0.3895
Ever taken a COVID-19 vaccine (yes)	756	88.1	707	87.8	49	92.5	0.3134
Worry about becoming (re)infected by the coronavirus							
Not at all	92	10.7	90	11.2	2	3.8	0.5598
A little worried	293	34.2	274	34.0	19	35.9	
Moderately worried	281	32.8	262	32.6	19	35.9	
Very worried	125	14.6	116	14.4	9	17.0	
Extremely worried	67	7.8	63	7.8	4	7.6	
Impact of COVID-19 on food insecurity							
No change (insure - insecure)	22	2.6	21	2.6	1	1.9	0.3098
Worse	72	8.4	71	8.8	1	1.9	
Better	4	0.5	4	0.5	0	0.0	
No change (secure – secure)	760	88.6	709	88.1	51	96.2	

^†^Missing values (n=1 in Total (n=1 in singleton births)).

Regarding infant feeding practices (Table 2), approximately 97% of participants who had singleton or multiple births planned to breastfeed during pregnancy, but 85.4% of them reported breastfeeding in the previous 24 hours, and some also reported providing infant formula (24.8%), expressed human milk (17.6%), or complementary foods (10.5%). Bivariate analysis showed that those who had singleton births were more likely to report breastfeeding their infants directly from breast in the previous 24 hours (p < 0.05), whereas those with multiple births were more likely to report feeding their infants with expressed breast milk or infant formula (p < 0.01). In addition, the impact of COVID-19 on baby fed directly from breast was not associated with multiple births, but those who had multiple births were more likely to report that COVID-19 shortened or lengthened the duration of feeding expressed breast milk compared to those with singleton births (p < 0.05). The mean score of belief towards breastfeeding was 8.53 (SD = 1.91) and there was no difference between singleton or multiple births.

**Table 2:** Bivariate association between singleton/multiple births and Infant feeding and support factors.

	Total		Singleton Births		Multiple Births		p-value
	n	%	n	%	n	%	
Breastfeed intention during pregnancy (yes)	829	96.6	778	96.7	51	96.2	0.8700
**Infant Feeding practice**							
Baby fed directly from breast last 24 hours (yes)	733	85.4	694	86.2	39	73.6	**0.0116**
Expressed breast milk last 24 hours (yes)	151	17.6	133	16.5	18	34.0	**0.0012**
Infant formula last 24 hours (yes)	213	24.8	181	22.5	32	60.4	**<.0001**
Complementary foods last 24 hours (yes)	90	10.5	85	10.6	5	9.4	0.7957
**Completed stopped breastfeeding/giving express breast milk**							
Yes, Competed stopped breastfeeding	83	9.7	74	9.2	9	17.0	0.1578
No, still giving breastmilk/breastfeeding giving express breast milk	750	87.4	708	88.0	42	79.3	
Never initiated breastfeeding and expressing breast milk	25	2.91	23	2.86	2	3.8	
**Impact of COVID on baby fed directly from breast**							
Did/do not intend to feed	42	4.9	40	5.0	2	3.8	0.5361
Shorter than I intended	86	10.0	78	9.7	8	15.1	
The same duration as I intended	676	78.8	635	78.9	41	77.4	
Longer than I intended	54	6.3	52	6.5	2	3.8	
**Impact of COVID-19 on baby fed with expressed breast milk**							
Did/do not intend to feed	336	39.2	322	40.0	14	26.4	**0.0283**
Shorter than I intended	86	10.0	78	9.7	8	15.1	
The same duration as I intended	386	45.0	362	45.0	24	45.3	
Longer than I intended	50	5.8	43	5.3	7	13.2	
							
**Belief towards breastfeeding (0–12 scores) (Mean, SD)**	8.53	1.91	8.51	1.90	8.79	2.07	0.3026
							
**Support for postnatal infant feeding (Mark all that apply)**							
I receive no support for infant feeding	196	22.8	192	23.9	4	7.6	**0.0062**
From healthcare professionals	490	57.1	458	56.9	32	60.4	0.6197
From spouse/partner, friend or relative	442	51.5	419	52.1	23	43.4	0.2221
From online support group (e.g., Facebook)	417	48.6	380	47.2	37	69.8	**0.0014**
Others	77	9.0	73	9.1	4	7.6	0.7074
**Methods of contacts with healthcare professionals for postnatal breastfeeding support (Mark all that apply)**							
Never	293	34.2	278	34.5	15	28.3	0.3541
In person	360	42.0	334	41.5	26	49.1	0.2796
By phone	350	40.8	332	41.2	18	34.0	0.2962
Video	102	11.9	93	11.6	9	17.0	0.2369
Others	43	5.0	39	4.8	4	7.6	0.3824
**Experience of any difficulties when receiving support via video contact among those who made a video contact (Mark all that apply)**							
I have not experienced any difficulties with using video contact for breastfeeding support	44	43.1	40	43.0	4	44.4	0.9339
The supporter was unable to clearly see the baby’s latch on my breast	47	46.1	44	47.3	3	33.3	0.4218
The supporter was unable to hear me well	7	6.9	6	6.5	1	11.1	0.5975
I cannot hear the supporter well	7	6.9	7	7.5	0	0.0	0.3937
I cannot see the supporter well	7	6.9	6	6.5	1	11.1	0.5975
I cannot operate the electronic device and breastfeed at the same time	23	22.6	22	23.7	1	11.1	0.3898
Others	7	6.9	5	5.4	2	22.2	0.0563
**Social support (MSSS)**							
Low/Medium social support (<25 scores)	199	23.2	185	23.0	14	26.4	0.5662
High social support (25–30 scores)	659	76.8	620	77.0	39	73.6	

The majority of participants with singleton or multiple births received high social support (76.8%) in Table 2. In terms of postnatal infant feeding support, approximately 50% of participants with singleton or multiple births received postnatal infant feeding support from healthcare professionals, spouses/partners/friends/relatives, or online support group. There was no significant difference in support from health professionals, spouses/partners/friends/relatives between singleton and multiple births, but multiple births were more likely to report receiving support from online support groups during the COVID-19 (p < 0.01). Meanwhile, about 23% reported receiving no support at all for infant feeding during the COVID-19, a higher rate in singleton than in multiple births (p < 0.01).

The most common methods of contacting healthcare professionals for postnatal breastfeeding support were ‘in person (42%)’ followed by ‘by phone (40.8%),’ and ‘video (11.9%)’, but 34.2% said that they never had contacts. Among those who had video contacts for breastfeeding support, 46.1% answered that the supporter could not clearly see the baby latching on, and 43.1% answered that they experienced no difficulties when receiving breastfeeding support video. However, there was no significant difference between singleton and multiple births in terms of (a) method of contacts with a healthcare professional for breastfeeding support and (b) experiencing difficulties when receiving breastfeeding support via video calls.

Multiple logistic regression results on associated factors with multiple births are presented in Table 3. Women with multiple births were significantly associated with their babies fed with infant formula in the last 24 hours (AOR 6.17, 95% CI 2.29–16.57), preterm birth (AOR 5.83, 95% CI 1.99–17.11), and caesarean birth (AOR 2.97, 95% CI 1.27–6.97), and birthweight (AOR 13.16, 95% CI 4.31–40.20 for less than 2.5 kg and AOR 0.11, 95% CI 0.02–0.51 for more than 3.5 kg). Other factors, such as NICU admission, postnatal depression, breastfeeding at breast or expressing breast milk to the baby, the impact of COVID-19 on expressed breast milk, not receiving support for infant feeding, and receiving postnatal infant feeding support from online support groups (e.g., Facebook) were not significantly associated.

**Table 3:** Multiple logistic regression analysis of associations between selected factors and multiple births.

	AOR (95% CI)
Preterm birth (yes vs no)	**5.83 (1.99 - 17.11)**
Birthweight (<2.5 kg vs 2.5–3.5 Kg)	**13.16 (4.31 - 40.20)**
Birthweight (>3.5 kg vs 2.5–3.5 Kg)	**0.11 (0.02 - 0.51)**
Mode of birth (caesarean vs vaginal)	**2.97 (1.27 - 6.97)**
Probable postnatal depression (yes vs. no)	0.41 (0.14 - 1.22)
NICU admission (yes)	1.00 (0.33 - 3.07)
Baby only fed directly from breast last 24 hours (yes vs. no)	1.57 (0.46 −5.32)
Expressed breast milk last 24 hours (yes vs. no)	1.91 (0.72 - 5.08)
Infant formula last 24 hours (yes vs. no)	**6.17 (2.29 - 16.57)**
Impact of COVID-19 on baby fed with expressed breast milk	
Not intended to feed vs. Longer	2.28 (0.81 - 6.42)
Shorter than intended vs. Longer	2.81 (0.75 - 10.48)
The same duration as I intended vs. Longer	0.47 (0.10 - 2.28)
I received no support for infant feeding (yes vs. no)	0.30 (0.07 - 1.38)
I received postnatal infant feeding support from online support group (e.g., Facebook) (yes vs. no)	2.08 (0.77 - 5.61)

Variables with p-value <0.05 from Chi-square tests were included.

Hosmer and Lemeshow Goodness-of-Fit Test = 10.2390 (p = 0.2486).

## Discussion

Of the 858 eligible participants, 53 (6.2%) experienced multiple births. Out of 640,209 registered births in England and Wales in 2020, 9,656 (1.5%) were multiple births [2]. This demonstrated our sampling size was substantially larger than the population, and that may be because recruitment of participates included via groups which supported families with multiple births. It is important to note that this study cannot attribute differences between multiple and singleton births solely to the COVID-19 pandemic, as no pre-pandemic comparator group was available. Our findings therefore provide cross-sectional associations observed during the pandemic rather than causal inference due to COVID-19 pandemic.

As with the UK national infant feed survey finding [22], our result showed that participants with multiple births were more likely to use infant formula than those with singleton births The physical and emotional challenges of breastfeeding more than one baby, as well as pressure for formula feed from hospital staff [23] might contribute to this finding. Moreover, whilst it was not clear whether those who answered ‘yes’ to this question had been using infant formula exclusively, or as a supplement to breastmilk, it might suggest that they felt it was not possible to sustain their babies’ nutritional needs on breastmilk alone [12]. However breastmilk lactated for multiple births has been shown to be higher in proteins and lower in lactose, when compared to samples taken from women with singleton births [24]. Additionally, when compared to samples collected from parents of multiple births with higher gestational age, breastmilk produced for extremely preterm babies from multiple births contained a significantly higher protein concentration. It was established this could maintain growth and organ development for children with low birthweight [24]. These findings may inform future policy and clinical guidelines, particularly in preparing tailored support for families with multiple births.

Further investigation is required as to whether those who intended to breastfeed wished to do this without using supplementary infant formula and what was needed to achieve this. A Cochrane review [25] found neither evidence from randomised controlled trials on the effectiveness of breastfeeding education and support for women with twins or higher order multiple births, nor the most successful way to provide support. It was not established what would be the best way to deliver the intervention or the timing of care. Current NICE guidelines address the challenges faced by those experiencing multiple pregnancies and differentiate their advice from singleton pregnancies [4]. However, this only pertains to antenatal care and there is a lack of recommendations specifically for postnatal care of those with multiple births. NICE [4] recommends referral to an enhanced team which includes an infant feeding specialist, not as a matter of routine but based on perceived need. However, it also recognized the need for research to be conducted specifically into breastfeeding support for families with multiple births [13]. Considering the obstetric factors associated with multiple births, lactation specialists should be included as members of the multidisciplinary team for all those with multiple births as routine care and not simply when needed. Moreover, future research could take a co-design approach to develop the intervention to support families with multiple births to breastfeed, followed by a robust randomised controlled trial to investigate the effectiveness of the intervention.

Timing of our data collection (July -November 2021) could be a reason that there were no statistically significant results in COVID-19 related factors from our multiple logistic regression analysis as participants were more adjusted to living with COVID-19, reducing the effects of COVID-19 related factors. On the other hand, the positive and negative impact of COVID-19 on breastfeeding, which has been found in other studies [17] has been demonstrated in our participants with multiple births feeding their baby with expressed milk shorter than intended, while also longer than they intended. Having more time at home during COVID-19 [17] might have encouraged feeding directly from the breast and/or expressing breast milk. However, lack of support, including how to express milk, might have turned some women to formula feeding [17]. It has also been found that online group support (e.g., Facebook) is valuable to people multiple births [23]. Virtual breastfeeding support is useful in its convenience and helps breastfeeding practices [26], but its effectiveness when compared to face-to-face consultation is unclear. Future investigation into this technology as a way to provide breastfeeding support could help inform guidance on utilising media and digital technology for future pandemics.

Mahajan et al. [27] found women with multiple births infected with COVID-19 in India had 8 times higher chance of preterm birth when compared to singleton births. This highlighted the high likelihood of those with multiple births having preterm birth [6] as also shown in our finding. This could be due to the presence of more than one foetus in the uterus that puts more physical and physiological stress on the pregnant woman’s body, increasing the possibility of complications leading to premature birth, which is a leading cause of infant morbidity and mortality in the UK [28]. Interventions to reduce the adverse outcomes of preterm birth such as feeding difficulties and maternal problems including anxiety, fatigue and flashbacks [29] should be considered for inclusion in routine maternal support for those with multiple births.

Our result showing caesarean birth was significantly more common in multiple births is consistent with a previous study during COVID-19 in Germany [30]. Due to various complications including high risks of preterm birth, excessive weight gain, placental abruption [31], multiple pregnancies were more likely to result in caesarean birth [5]. Although caesarean birth can be lifesaving, it presents long-term health consequences for women and babies. For example, there were increased risks of miscarriage, still birth, placenta previa, and placental abruption with pregnancies following caesarean birth [32]. Children born by caesarean birth had higher risks of asthma up to age 12 and obesity up to age 5 [32].

While our study did not investigate correlation between the participants who had caesarean birth and those who reported using infant formula, previous research has suggested that the planned mode of birth in multiple births (vaginal vs caesarean birth) did not affect the initiation or continuation of breastfeeding [33]. However, a literature review [34] reported that when compared with vaginal birth, caesarean birth can result in delayed breastfeeding and earlier cessation of exclusive breastfeeding citing physical limitations after surgery, wound pain, anxiety, and breastfeeding inability as predominant barriers. Alongside the higher likelihood of preterm birth and caesarean birth, those with multiple births had higher odds of babies born with birthweight less than 2.5 kg (low birthweight) also concurred with previous research [7,8]. Having babies born with low birthweight was found to increase the women’s risk of having later Type 2 diabetes and cardiovascular disease later in life [35]. Babies born with low birthweight had higher risks of long-term neurologic disability, impaired academic and language development, and cardiovascular disease and diabetes [36]. Babies who fall into this weight category have higher nutritional requirements to ensure they reach the same growth milestones as babies born in higher weight categories and lower their risk of adverse health outcomes [37], and therefore effective feeding support is essential. These results suggested a need for additional support regarding infant nutrition for multiple births.

### Strengths and limitations

An essential strength of this work is its originality, being the first of its kind in the UK and can inform support needs of those with multiple births. Additionally, the proportion of multiple births included in this study was larger than that of the population. Our findings, despite during the COVID-19, highlighting the essential factors that are associated multiple births, have supported our recommendations for healthcare policies/guidelines and practices that need to be considered for a future pandemic and beyond to differentiate them from standard services.

Nevertheless, the use of convenience sampling and online recruitment limited the representativeness of study population. Our results may not be generalizable and there may be selection bias favouring certain groups, e.g., those who had easy online access. As the survey was online, certain demographics might be excluded such as those with limited or no internet access. There might be social desirability bias with the self-reported data (e.g., COVID-19 infection status, COVID-19 vaccination uptake, infant feeding practices) which might affect the reliability of some findings. As this is a cross-sectional study, causal relationships cannot be established. The relatively small number of multiple-birth participants (n = 53) may limit the stability of logistic regression models and increase the possibility of type II error by reducing the statistical power of the model to detect true effects. A larger proportion of multiple births in this study may provide more insights on feeding practices and associated factors. We did not know the relationships between participants’ COVID-19 infection status during the time of birth and the obstetric factors. Therefore, whether the factors associated with multiple births were related to being infected with COVID-19 at birth cannot be established. Finally, the absence of baseline pre-pandemic data due to cross-sectional data collected during the COVID-19 pandemic prevents conclusions about whether COVID-19 directly modified outcomes for families with multiple births.

## Conclusions

The study’s findings highlighted that formula feeding, preterm birth, caesarian birth, and birthweight were associated with multiple births during COVID-19 in the UK. This suggests that those with multiple births require tailored breastfeeding support which integrates lactation specialists into their care. Leveraging the use of technology in remote breastfeeding support for those with multiple births is recommended. Further research into effective interventions is needed to improve exclusive breastfeeding rates amongst those with multiple births. Future research should be conducted to investigate how technology can be improved for remote breastfeeding support during and beyond a pandemic.
